# Toxicity of the organophosphate insecticide sumithion to embryo and larvae of zebrafish

**DOI:** 10.1016/j.toxrep.2020.02.004

**Published:** 2020-02-07

**Authors:** Mohammad Shadiqur Rahman, S M Majharul Islam, Anamul Haque, Md. Shahjahan

**Affiliations:** Laboratory of Fish Ecophysiology, Department of Fisheries Management, Bangladesh Agricultural University, Mymensingh 2202, Bangladesh

**Keywords:** Organophosphate insecticide, Acute toxicity, Zebrafish, Embryo, Larvae

## Abstract

Sumithion, a synthetic organophosphate, is widely used as an agricultural insecticide and for control of tiger bug (*Cicindela* spp.) in larval rearing for aquaculture. An experiment was conducted to examine the effects of sumithion on embryological and larval development of zebrafish *Danio rerio*. Fertilized egg (n = 100) and larvae (n = 100) were exposed to six concentrations of sumithion (0, 0.1, 0.2, 0.4, 0.8 and 1.6 mg L^−1^) in three replicates. LC_50_ values for embryos and larvae were calculated by probit analysis. The 24 h LC_50_ value of sumithion for embryo was 0.235 (0.079-0.428) mgL^−1^. Increasing sumithion concentrations decreased hatching success and increased embryonic mortality. In embryos, sumithion induced several malformations including immature yolk sac, dark yolk sac, yolk sac bud, broken eggshell and notochord, unhatched eggs. Larval LC_50_ values at 24, 48 and 72 h of various doses of sumithion exposure were 0.620 (0.436-0.963), 0.475 (0.302-0.801) and 0.341 (0.177-0.617) mgL^−1^, respectively. Various physical deformities, including edema, notochord deformity, yolk sac damage, body arcuation, lordosis and black pigmentation on the yolk sac were evident in response to different concentrations of sumithion. The results of the current study indicate that sumithion exerts developmental toxicity to zebrafish embryos and larvae. It is expected that current findings will increase sensitivity about the toxic effect of sumithion in early development, as well the possibility of similar actions induced by other insecticides and pesticides.

## Highlights

• The 24 h LC_50_ value of sumithion for embryo was 0.235 mg L^−1^.
• Several malformations were observed in embryos exposed to different concentrations of sumithion.
• The 24, 48 and 72 h LC_50_ values of sumithion for larvae were 0.620, 0.475 and 0.341 mg L^−1^, respectively.
• Different deformities were evident in the sumithion exposed larvae.

## Graphical abstract

**Figure:**
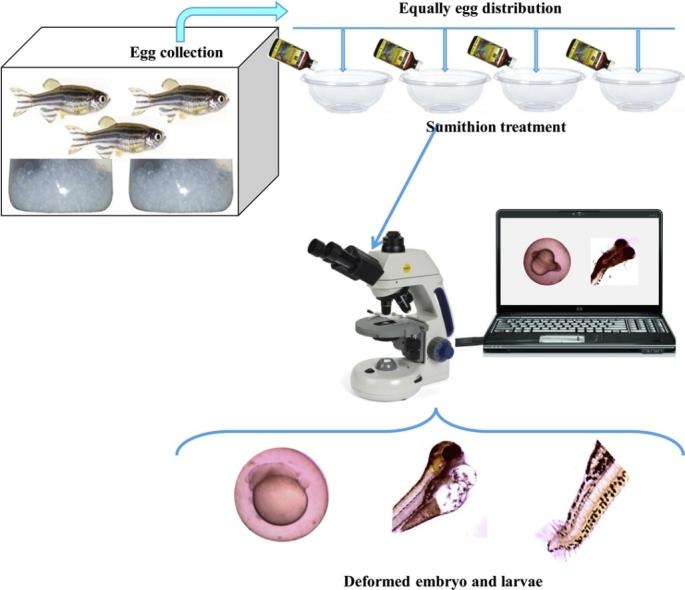

## 1 Introduction

Indiscriminate use of pesticides and insecticides for increased crop produciton in Bangladesh [1] has increased sharply, from applications of 7350 metric ton (MT) of such agents in 1992 to 45,172 metric ton (MT) in 2010 [2]. These pesticides and insecticides reach the aquatic environment through surface runoff, spray drift, precipitation or direct deposition [3]. Among several types of pesticides and insecticides, the organophosphate pesticide sumithion, O, O Dimethyl O-(3-methyl-4 nitrophenyl) is used extensively in Bangladesh. It has been reported that 0.02 μg/L of sumithion was found in the water of Biwa Lake in Japan [3]. It is applied for the control of a viariety of insects and pests in rice, vegetables, fruits, cereals, cotton etc. [5]. In addition, it is also used in public health program to control flies, mosquitos and cockroaches. In addition, it is used to eliminate tiger bug (*Cicindela* spp.) in aquaculture ponds, although sumithion is reportedly toxic fish and other aquatic organisms [6]. The pesticides and insecticides in aquatic environments can affect aquatic organisms through neuro-behavior and other physiological mechanisms [[7], [8], [9], [10], [11], [12]], resulting in sharp reductions of fish production. For these reasons, the study of toxicity of pesticides and insecticides on aquatic organisms like fish is critically important.

Pesticides and insecticide toxicity can be assessed by the quantitative study of early development in aquatic organisms [13,14]. Fish embryos and larvae are extremely sensitive to environmental pollutants [13], which can result in morphological changes, and such biomarkers can be used to assess the influences of pollutants on aquatic animals [15]. Negative effects of bioactive materials like buprofezin (5–100 mg L^−1^) and endosulfan (96 h LC50 0.22 mg L^−1^) on early juvenile stages of fish have been measures, e.g. the reduced hatching success of African catfish embryos [16,17]. Similarly, cypermethrin (400 μgL^−^¹) caused quantifiable malformations to embryos and larvae of zebrafish [18] and banded gourami [13].

The zebrafish (*Danio rerio*) is a small cyprinid fish from the Ganges and Brahmaputra river basins in India, Bangladesh and Myanmar [19]. This is an aquarium fish species, and an increasingly popular and versatile laboratory. In the research field of genetics, neurophysiology, biomedicine and developmental biology, this fish species is universally used as vertebrate model animals [20,21]. Several studies reported that this fish species also used as model animal to study the impacts of environmental threats [[22], [23], [24]]. Embryos of this fish are comparatively big, strong and clear, and rapid embryonic development [25]. This fish is also patently parallel to mammalian models and human toxicity testing, exhibiting a diurnal sleep cycle with similarities to mammalian sleep behavior [26]. It has been reported that sumithion (1.0 mg L^−1^) altered the blood glucose level and histo-architecture intestine in adult zebrafish [27,28]. Although there are some studies on toxicity of sumithion in fish species [[10], [11], [12], [13], [14]], no studies have focused on the toxicity of sumithion on early development of zebrafish. Therefore, the present study was intended to assess the toxicity of sumithion on embryonic and larval zebrafish. The findings of this investigation will be useful for the understanding of negative aquatic enviornmental impacts of pesticides and insecticides, especially on fishes. It may be possible to save fishes by controlling environmental pollution by pesticides and insecticides, and by refining the strategies for use of sumithion in larval rearing aquaculture ponds.

## 2 Materials and methods

### 2.1 Collection of experimental fish

Wild-type adult zebrafish were collected from different ponds neighboring to the Faculty of Fisheries building, Bangladesh Agricultural University. Total length and weight of fishes ranged from 3 to 5 cm and 0.7–1.2 g, respectively. The fishes were reared in the aquaria and fed twice a day on a commercially prepared diet. The water quality parameters, such as temperature (30 ± 05⁰C), pH (7.52 ± 0.09), dissolved oxygen (6.08 ± 0.19 mg L^−1^), free CO_2_ (6.38 ± 0.48 mg L^−1^) and total alkalinity (194.25 ± 9.95 mg L^−1^) were recorded to be in the optimum range during the rearing period. The Animal Welfare and Ethical Committee, Bangladesh Agricultural University approved the experimental procedures used in this study.

### 2.2 Collection of pesticide

The organophosphate Sumithion 50EC, O, O Dimethyl O-(3-methyl-4-nitrophenyl) was used in this experiment. It is availbale locally in liquid form as a commercial insecticide with 500 g L^−1^ of fenitrothion as the active ingredient. The selected concentrations of sumithion were prepared as per EC percentage and carefully transferred into test bowls containing de-chlorinated tap water.

### 2.3 Collection of fertilized eggs

Collected fishes were stocked in six aquaria in equal numbers (50) per each aquarium. The ratio of male and female (1:1) was maintained. Some marble with plastic Petri dishes and artificial trees were placed on the bottom of each aquarium. The zebrafish were spawned early in the morning and eggs were deposited in between the gaps of marbles in the plastic Petri dishes. Just after spawning, fertilized eggs were collected from the Petri dishes using eye-droppers in this study.

### 2.4 Effects of sumithion on embryonic and larval development

A total of 100 fertilized egg were distributed into 18 prior arranged sets of plastic bowls containing six different concentrations of sumithion (0, 0.1, 0.2, 0.4, 0.8 and 1.6 mg L^−1^), each of which executed with three replicates. The different concentrations of pesticide were renewed at every 24 h intervals to maintain the same concentrations during the study period. Dead embryos were identified as white opaque color and not responding to agitation of water by plastic spoon. The incubation duration and hatching success were noted for control and all treated groups. Dead embryos were counted and recorded after 24 h of exposure. Similarly, 100 larvae were exposed in 18 previously-prepared sets of plastic bowls containing six different concentrations of sumithion (0, 0.1, 0.2, 0.4, 0.8 and 1.6 mg L^−1^) each with three replicates. Records of mortality of larvae were made at logarithmic time intervals (24, 48, 72, and 96 h) from the beginning of the exposure. Deformities of embryos and larvae were perceived and snapped at 6 h and 12 h intervals, respectively under a microscope (MICROS MCX 100, Austria) connected with a digital camera (Magnus analytics, Model-MIPS) in a computer.

### 2.5 Data analysis

Data of hatching and mortality of embryos and larvae were presented as mean ± standard deviation (SD). The LC_50_ values were calculated using probit analysis. Data were analyzed by one-way analysis of variance (ANOVA) followed by Tukey’s post-hoc test to assess the statistical significance of differences among responses to treatments. Statistical significance was set at the p < 0.05 level. Statistical analyses were performed using PASW Statistics 18.0 software (IBM SPSS Statistics, IBM, Chicago, USA).

## 3 Results

### 3.1 Toxicity of sumithion to embryos of zebrafish

**Fig. 1:**
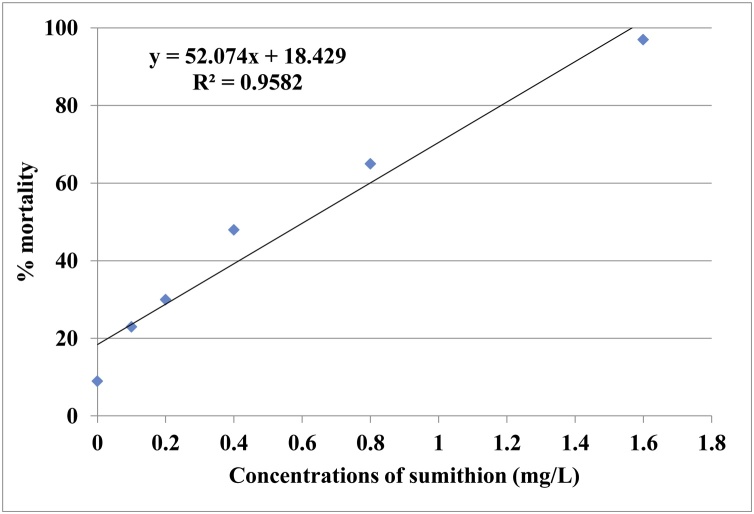
The linear transformation and relationship of probit of concentrations of sumithion used to determine LC_50_ values for embryos after 24 h of exposure to sumithion.

**Fig. 2:**
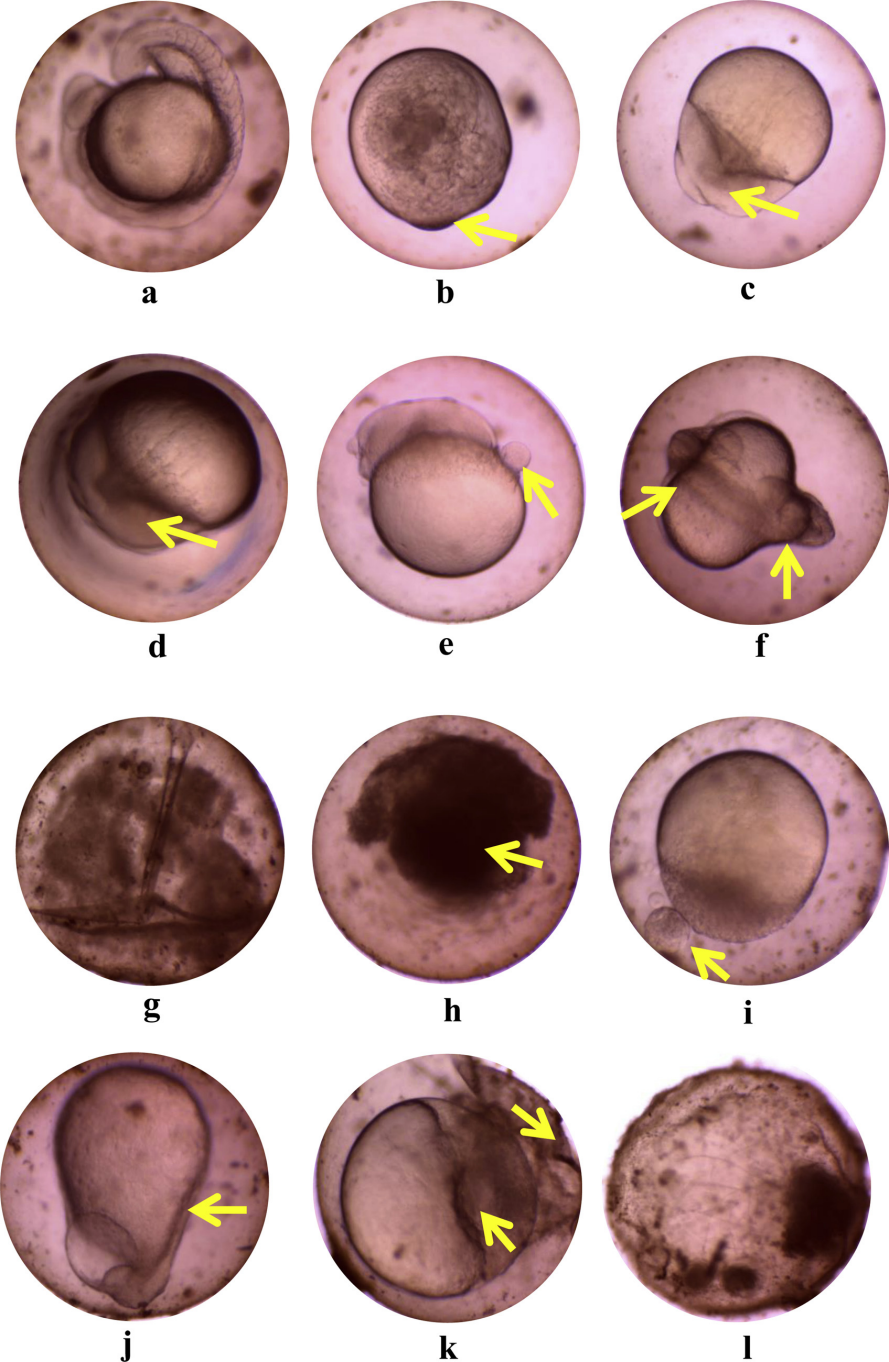
Deformity observed in embryos of zebrafish exposed to sumithion. a. normal embryo after 12 h of exposure to 0 mg L^−1^ of sumithion; b. lack of somite formation after 30 min of exposure to 0.2 mg L^−1^ of sumithion; c. immature yolk sac after 2 h and 30 min of exposure to 0.2 mg L^−1^ of sumithion; d. yolk sac damaged after 2 h and 15 min of exposure to 1.6 mg L^−1^ of sumithion; e. yolk sac bud/snowball after 2 h and 30 min of exposure to 0.8 mg L^−1^ of sumithion; f. shield after 4 h of exposure to 0.2 mg L^−1^ of sumithion; g. unhatched segmentation after 5 h of exposure to 0.2 mg L-1 of sumithion; h. dark yolk sac after 5 h and 15 min of exposure to 1.6 mg L^−1^ of sumithion; i. swelling/pustule after 5 h and 30 min of exposure to 0.4 mg L^−1^ of sumithion; j. yolk sac elongated shape after 11 h of exposure to 1.6 mg L^−1^ of sumithion; k. egg shell broken and yolk sac damaged after 11 h of exposure to 1.6 mg L^−1^ of sumithion; l. dead egg after 24 h of exposure to 1.6 mg L^−1^ of sumithion.

With increasing concentrations of sumithion, the incubation periods were extended in the embryonic development of zebrafish. There was a significant (p < 0.05) increase in mortality of embryos and significantly (p < 0.05) decrease in hatching success in response to increasing sumithion concentrations (Table 1). The 24 h LC_50_ values of sumithion for zebrafish embryo was 0.235 (0.079-0.428) mgL^−1^. Fig. 1 showed the linear transformation of percentage mortality of embryos and concentrations of sumithion. Several deformities, such as immature yolk sac, dark yolk sac, yolk sac bud, broken eggshell and notochord, unhatched eggs etc. were observed in embryos after exposure to different concentrations of sumithion (Fig. 2). Although few abnormalities were observed in in 0.2 mg L^−1^ of sumithion, most of the abnormalities were found when exposed to 0.4–1.6 mg L^−1^ of sumithion. No abnormalities were observed after exposure to less than 0.2 mg L^−1^ concentrations of sumithion.

**Table 1:** Toxicity of sumithion on the embryo of zebrafish (n = 100 embryos).

Concentrations (mg/L)	Incubation period (h)	Number of dead embryos at 24 h	Hatching Success (%)
0.0	45.10	9.0 ± 1.6	91.50
0.1	52.30	33.0 ± 2.6*	83.50
0.2	55.30	47.0 ± 3.2*	66.50*
0.4	54.00	53.0 ± 5.6*	48.50*
0.8	58.30	77.0 ± 7.5*	21.50*
1.6	66.10	97.0 ± 6.0*	3.50*
LC50 value		0.235 (0.079-0.428)	

* Significance level (p < 0.05).

### 3.2 Toxicity of sumithion to larvae of zebrafish

**Fig. 3:**
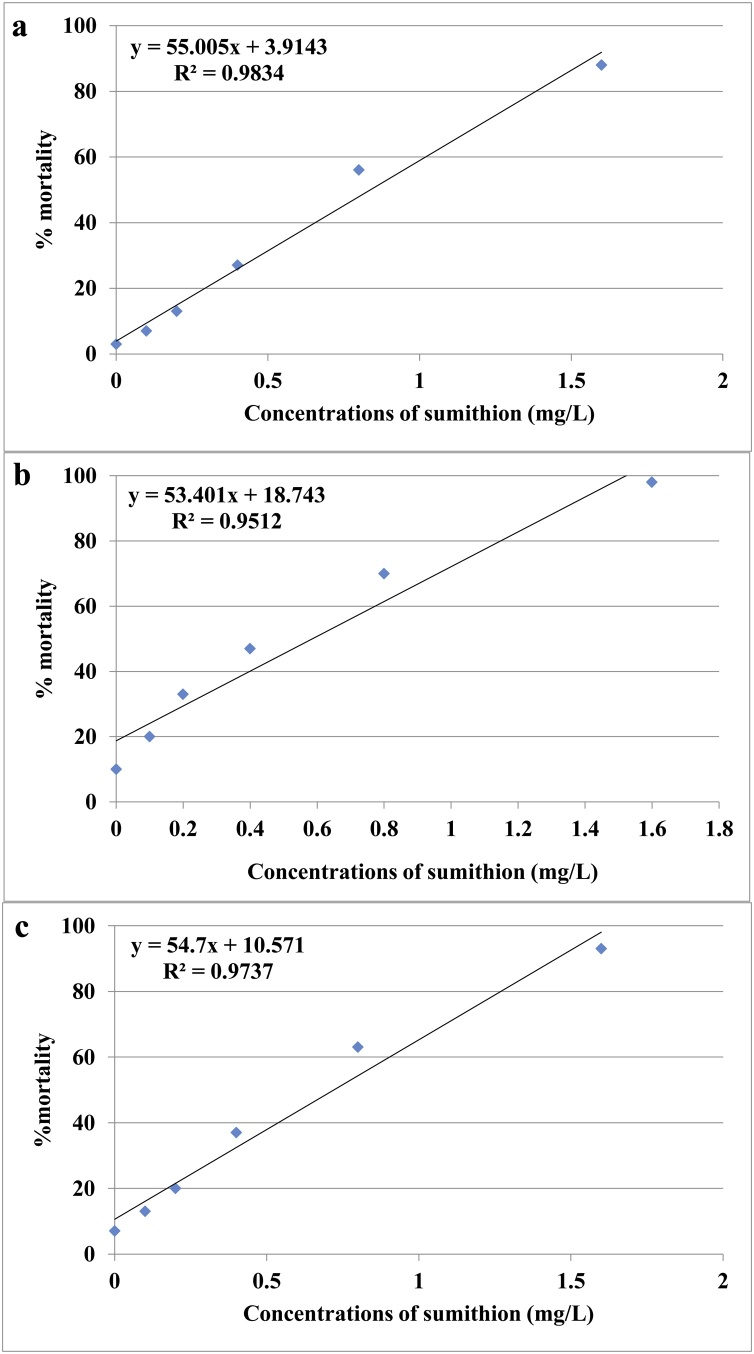
The linear transformation and relationship of probit of concentrations of sumithion used to determine LC_50_ values for larvae after (a) 24, (b) 48 and (c) 72 h of exposure to sumithion.

**Fig. 4:**
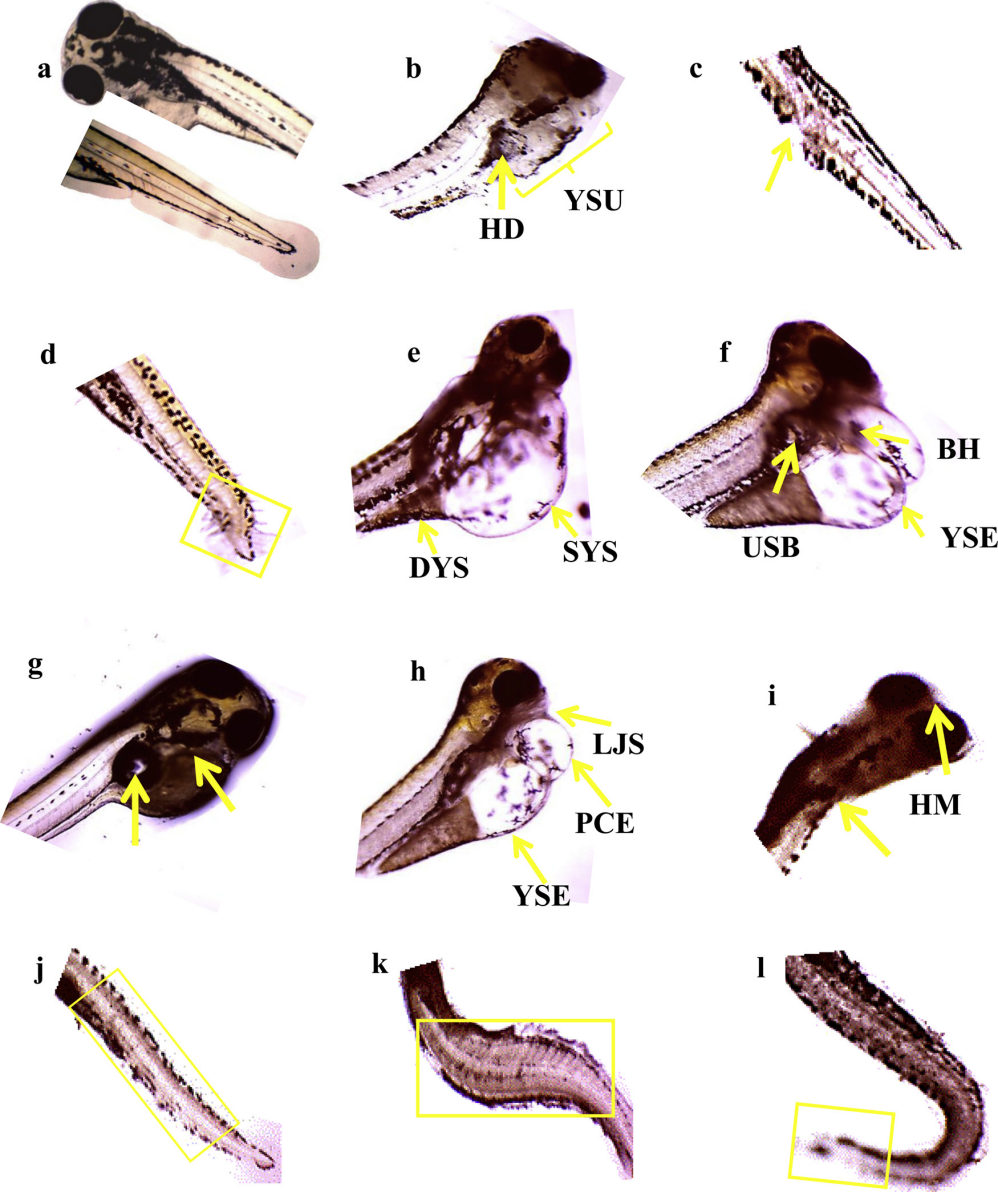
Deformity observed in larvae of zebrafish exposed to sumithion. a. normal larvae; b. yolk sac ulceration (YSU) and heart damage (HD) after 36 h of exposure to 0.4 mg L^−1^ of sumithion; c. lesion/ulceration of caudal region after 36 h of exposure to 0.8 mg L^−1^ of sumithion; d. end tail shortening and malformation after 36 h of exposure to 0.8 mg L^−1^ of sumithion; e. swollen yolk sac (SYS), swollen and discontinuous yolk sac (DYS) after 48 h of exposure to 1.6 mg L^−1^ of sumithion; f. uninflated swim bladder (USB), yolk sac edema (YSE), blood hemorrhage (BH) after 60 h of exposure to 0.8 mg L^−1^ of sumithion; g. black pigmentation on yolk sac and unlooped heart after 72 h of exposure to 0.8 mg L^−1^ of sumithion; h. pericardial sac edema (PSE), lower jaw shortening (LJS), yolk sac edema (YSE) after 60 h of exposure to 1.6 mg L^−1^ of sumithion; i. head malformation (HM) and spine scoliosis after 84 h of exposure to 0.4 mg L^−1^ of sumithion; *J*; notochord abnormalities after 84 h of exposure to 0.8 mg L^−1^ of sumithion; k. lordosis and irregular caudal region after 96 h of exposure to 0.4 mg L^−1^ of sumithion; l. deformed posterior part of body and tail ulceration after 96 h of exposure to 0.4 mg L^−1^ of sumithion.

Mortality of larvae at 24, 48 and 72 h increased significantly (p < 0.05) in response to increasing concentrations of sumithion (Table 2). The 24, 48 and 72 h LC_50_ values of sumithion for zebrafish larvae were 0.620 (0.436-0.963), 0.475 (0.302-0.801) and 0.341 (0.177-0.617) mgL^−1^, respectively. The linear transformation of percentage mortality of larvae and concentration of sumithion are showed in Fig. 3a, b and c for 24, 48 and 72 h, respectively. Abnormalities were also evident in zebrafish larvae, for example deformed and broken notochord, uninflated swim bladder, yolk-sac edema, pericardial sac edema, body arcuation, lordosis, scoliosis and irregular caudal region after exposure to various concentrations of sumithion (Fig. 4). Most of the deformities in larvae were found when exposed to 0.4 to 1.6 mg L^−1^ of sumithion. There was no noticeable malformation in larvae exposed to <0.4 mg L^−1^ concentrations of sumithion.

**Table 2:** Toxicity of sumithion on the larvae of zebrafish (n = 100 larvae).

Concentrations (mg/L)	Number of dead larvae at 24 h	Number of dead larvae at 48 h	Number of dead larvae at 72 h
0.0	3.0 ± 0.6	7.0 ± 0.6	10.0 ± 1.6
0.1	7.0 ± 1.6	13.0 ± 1.6	20.0 ± 2.6
0.2	13.0 ± 4.0*	20.0 ± 4.0*	33.0 ± 3.6*
0.4	27.0 ± 4.6*	37.0 ± 4.6*	47.0 ± 6.0*
0.8	56.0 ± 7.6*	63.0 ± 7.6*	70.0 ± 5.6*
1.6	88.0 ± 1.4*	93.0 ± 1.4*	98.0 ± 1.6*
LC50 value	0.620 (0.436-0.963)	0.475 (0.302-0.801)	0.341 (0.177-0.617)

* Significance level (p < 0.05).

## 4 Discussion

Extensive usage of pesticides and insecticides are problematic because of their unsafe effects on non-target organisms like fish. Hence we observed a range of impacts of sumithion to embryos and larvae of zebrafish. The incubation time, hatching rate and survivability of embryos and larvae were affected after exposure to different concentrations of sumithion. A variety of serious developmental embryonic and larval deformities were recorded during the study period.

In the present investigation, the hatching success remarkably decreased with increasing concentrations of sumithion. Earlier reports showed that pesticides have negative impacts on the hatchability of several fishes. For example, there was a significant decrease in hatching rate of zebrafish embryos after exposure to different concentrations of dimethoate [29] and alphamethrin [30]. Similar results were reported in common carp embryos [31] and turbot eggs [32] after exposure to different concentrations of diazinon. Significant decreases in hatching success were also reported in the embryos of common carp exposed to pyrethroid deltamethrin [33], cypermethrin [34], cyhalothrin [35] and cyperkill [36]. Similarly, for African catfish embryos, lowered hatching rate was witnessed after exposure to various concentrations of buprofezin [17] and endosulfan [16]. Reduced hatchability might be endorsed to the hindered development of embryos as an important effect of the toxicant. It may be due to inhibition of the tetraspanib cd63 gene that caused deficiency in secretion of proteolytic enzymes essential for controlling of the chorion [37].

The prolonged incubation period observed after exposure to sumithion in the present study may be because of lowered oxygen or troubles of enzyme responsible for hatching. Usually, the chorion is digested by the proteolytic hatching enzyme secreted from hatching gland cells of embryo during the normal hatching process of fish embryos. Protease structure and function might be disrupted due to toxicants which block the pore canals of the chorions, resulting in oxygen shortages for the development of embryos [38]. Consequently, with increasing concentrations of sumithion significantly increased the mortality of embryos and larvae of zebrafish. Remarkably, the percentage of mortality was higher in embryos than larvae, indicating that embryos are more sensitive to sumithion toxicity to than are zebrafish larvae. The sensitivity of embryos and larvae to toxicants usually varies in a species-dependent fashion [30,39]. In the present study, the LC50 value at 24 h of sumithion for embryo was 0.235 mg L^−1^, while the LC50 values of sumithion for larvae at 24, 48 and 72 h were 0.620, 0.475 and 0.341 mg L^−1^, respectively. Similar results were reported for toxicity of deltamethrin for common carp larvae [33], rainbow trout fry [40], European catfish fingerlings [41] and spirlin (*Alburnoides bipunctatus*) larvae and fingerlings [42].

Several deformities in the embryos and larvae of zebrafish were evident after exposure to different concentrations of sumithion, especially in higher concentrations (Fig. 2, Fig. 4). Similar deformities were reported in zebrafish embryos and larvae exposed to different concentrations of cypemethrin [18], in African catfish following exposure to buprofezin [17], in banded gourami when exposed to chlorpyrifos [13] and in stinging catfish when exposed to sumithion [14]. It has been reported that different heavy metals exposure also causes deformities of larvae of zebrafish [43]. The present study is also supported by previous results on zebrafish exposed to malathion [44], fipronil [45], acetofenate [46], cartap [47], bifenthrin [48], chlorpyrifos [49,50] and endosulfan [51]. Formation of edema in embryos and post-hatch larvae was increased with increasing concentrations of sumithion, possibly be due to failure of osmoregulation associated with pesticide accumulations, or perhaps resulting from down regulation of pkt7 (a critical regulator of slc2a 10/glut 10) and wwox genes [52]. Other deformities like spinal curvature (lordosis, kyphosis and scoliosis) seen commonly in zebrafish embryos and larvae exposed to toxicants might result from differential accumulation of toxicants and lack of neuromuscular coordination. Moreover, spinal curvature might be the consequence of decreased collagens in the spinal column, changing amino acid composition [53] or due to down regulations of pkt7 gene, a critical regulator of wnt signaling [54].

The present conclusion is that dose-dependent toxic effects of sumithion on zebrafish greatly influence hatching success, survival and incubation period, and that sumithion also induces embryonic and larval physical malformations. Because contamination with sumithion is potentially harmful to aquatic environments, agricultural pesticide and insecticide use should be very carefully considered. Our study confirms that zebrafish have potential as a model animal to evaluate the developmental harmfulness of environmental pollutants. Further studies are recommended for understanding how sumithion affect juvenile and possibly adult zebrafish in the long-term of toxicity.

## Declaration of Competing Interest

The authors declare that they have no known competing financial interests or personal relationships that could have appeared to influence the work reported in this paper.
